# Supplementary material for: Molecular Dynamics and Docking Simulations of Homologous RsmE Methyltransferases Hints at a General Mechanism for Substrate Release upon Uridine Methylation on 16S rRNA
Source: Int J Mol Sci. 2023 Nov 24;24(23):16722. doi: 10.3390/ijms242316722 (PMC10706118; doi:10.3390/ijms242316722)
Supplement: Supplementary file 1 [file ijms-24-16722-s001.zip › ijms-2641595-supplementary.pdf]

International Journal of
*Molecular Sciences*

MDPI

**Supplement Material**

for "Molecular Dynamics and Docking Simulations of Homologous RsmE Methyltransferases Hints at a General Mechanism for Substrate Release upon Uridine Methylation on 16S rRNA"

by Aaron Hernández-Cid, Jorge Lozano-Aponte and Thomas Scior

*Receptors and ligands employed in Molecular Dynamics*

**Table S1.** Summary of receptors and ligands employed for simulations in this work. EM: Energy minimization. MD: Molecular Dynamics simulations. The crystal structures of SAM and SAH were taken from 5VM8 and 2EGW, respectively. The different methods were applied with RsmE monomers for the three species. BdD: Blind dock, BkD: Backdock.

| PDB code [Ref.] | Species | Receptor | Crystalized ligand | Ligands to evaluate | Computational analysis method |
|---|---|---|---|---|---|
| **5VM8** [18] * | *Neisseria gonorrhoeae* | RsmE | SAM | SAM (BkD), SAH (BdD) | Molecular docking, MD (RsmE + SAH) |
| **2EGW** [18] | *Aquifex aeolicus* | RsmE | SAH | SAM (BdD), SAH (BkD) | Molecular docking |
| **4E8B** [2] | *Escherichia coli K-12* | RsmE | None | SAM (BdD), SAH (BdD) | Molecular docking, EM-MD (RsmE + SAH) |

* Unpublished but cited in [18] from the Seattle Structural Genomics Center for Infectious Disease: Edwards, T.E., Conrady, D.G., Lorimer, D.D. Crystal structure of a Ribosomal RNA small subunit methyltransferase E (RsmE) from Neisseria gonorrhoeae bound to S-adenosyl methionine. To be published; https://doi.org/10.2210/pdb5vm8/pdb; last visit Sept. 6th 2023.

*The crystal structure of N. gonorrhoeae RsmE*

The structure of *N. gonorrhoeae* RsmE was deposited under the PDB identifier 5MV8 and determined to 2.40 Å resolution. This RsmE, as its counterparts, shows an overall structure consisting of two distinct domains, the PUA-like N-terminal (NTD) domain and the SPOUT-like C-terminal (CTD), the latter comprising residues 26 to 72 and the former residues 79 to 234, both separated by a short linker region (Fig. S1). The NTD contains a twisted sheet composed of five strands (β1-β5), which are typical of RsmE NTD, aligned antiparallel to each other and interleaved with a single helix (Fig. S1).

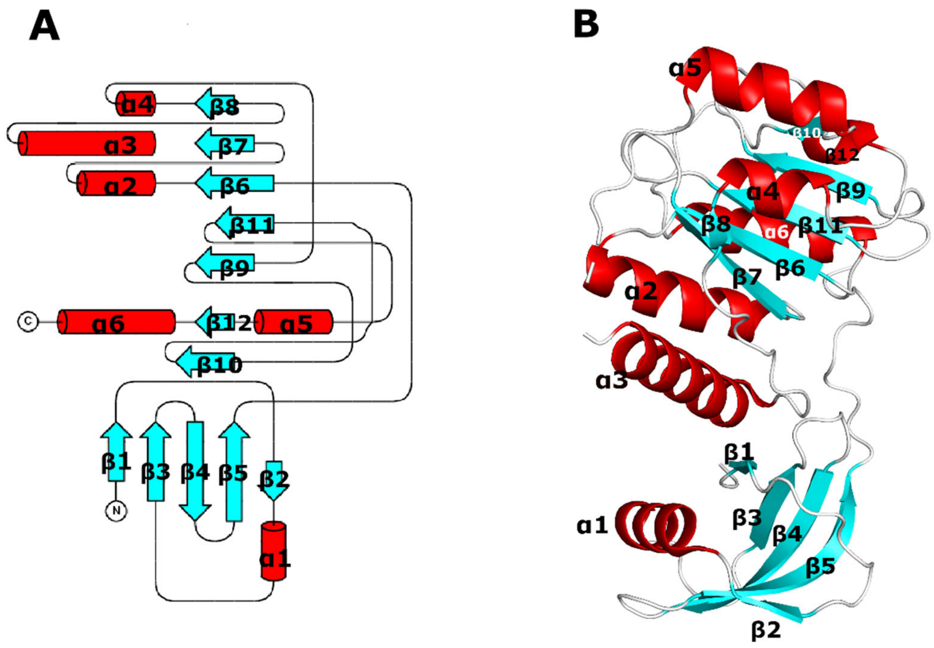


**Figure S1.** (**A**) Topological map of target proteins prepared in TopDraw. (**B**) The crystal structure of RsmE of *N. gonorrhoeae* from PDB entry 5MV8. PUA: 1-66, SPOUT: 71-241. CTD: C-terminal domain, NTD: N-terminal domain. The relative position of the catalytic site is shown with SAM. The protein structure was prepared with PYMOL.

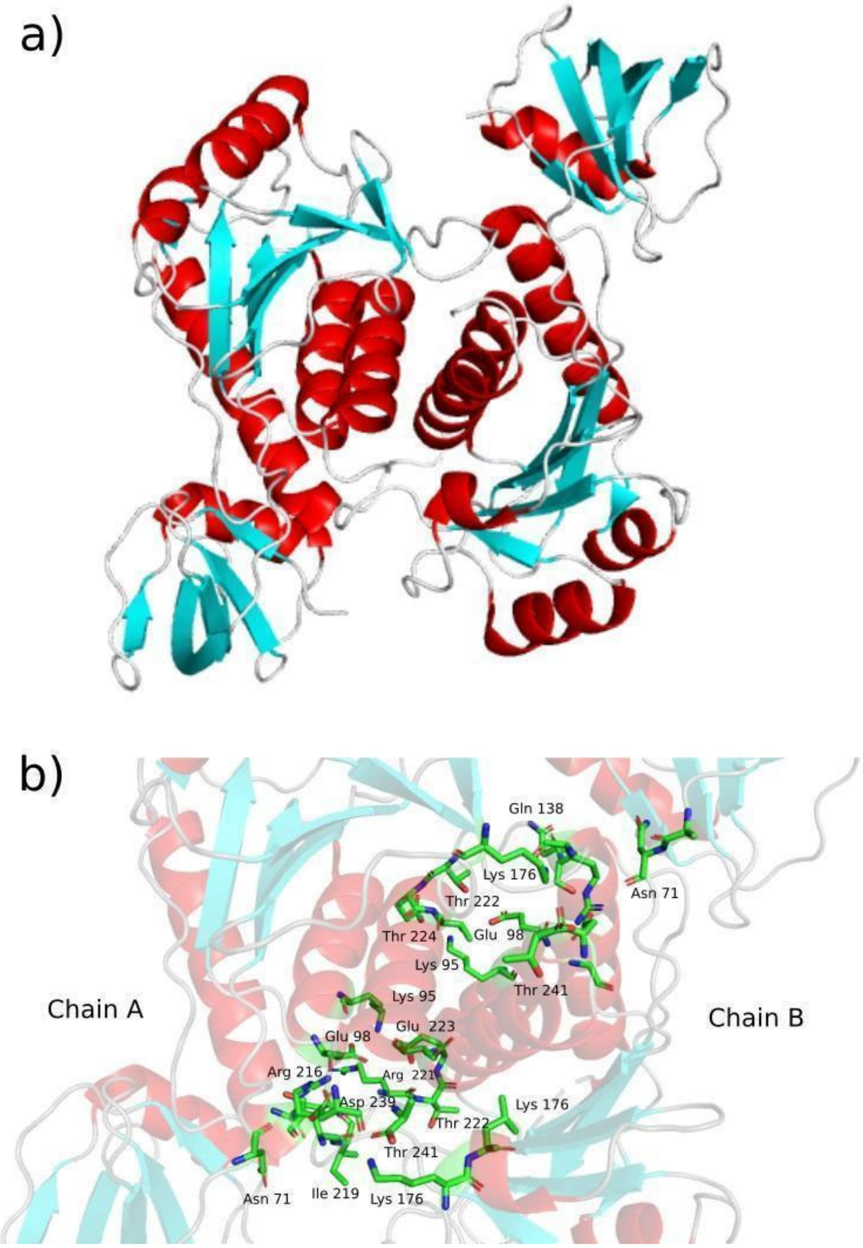


**Figure S2.** Target protein structures and catalytic sites. (**A**) The asymmetric unit of the RsmE crystal structure of *N. gonorrhoeae***.** (**B**) The dimer interface in the RsmE with the contact amino acids represented as sticks**.** Figures were prepared in Pymol using PDB 5mV8.

*Root mean squared deviation from superposition of the crystal RsmE.*

**Table S2.** Root mean square deviation values (RMSD by SPDBV [34]) measured by superposition of the crystal RsmE structures reported in PDB over 5VM8 structure (Ref.), and percentages of identity (% Id.) obtained by MSA of the corresponding UniProt sequences. Bb: backbone. The three species studied in this work are underlined (*Neisseria gonorrhoeae*, *Aquifex aeolicus* and *Escherichia coli*).

| **PDB code [Ref.]** | **Crystal structure of ligand** | **Species** | **RMSD Å (Bb atoms)** | **UniProt id.** | **% Id.** | **Color Fig. 2** |
|---|---|---|---|---|---|---|
| **1NXZ [21]** | - | *Haemophilus influenzae* | 1.38 (848) | P44627 | 41.25 | |
| **1V6Z [53]** | - | *Thermus thermophilus HB8* | 1.32 (240) | Q5SKI6 | 32.88 | |
| **1VHK [22]** | - | *Bacillus subtilis* | 1.20 (704) | P54461 | 27.62 | |
| **1VHY [22]** | - | *Haemophilus influenzae* | 1.40 (848) | P44627 | 41.25 | |
| **1Z85 [54]** | - | *Thermotoga maritima MSB8* | 1.43 (488) | R4NSV1 | 26.98 | |
| **2CX8 [55]** | SAH | *Thermus thermophilus* | 1.39 (232) | Q5SKI6 | 32.88 | |
| **2YXL [56]** | SAM | Pyrococcus horikoshii | | O58581 | | |
| **2EGV [18]** | SAM | *Aquifex aeolicus* | 1.35 (728) | O66552 | 29.86 | |
| **2EGW [18]** | SAH | *Aquifex aeolicus* | 1.37 (708) | O66552 | 29.86 | |
| **2Z0Y [57]** | SAM | *Thermus thermophilus* | 1.37 (232) | Q5SKI6 | 32.88 | |
| **3KW2 [58]** | Adenosine | *Porphyromonas gingivalis* | 1.42 (788) | B2RH75 | 28.88 | |
| **4E8B [2]** | - | *Escherichia coli K-12* | 1.39 (868) | P0AGL7 | 39.33 | |
| **4J3C [59]** | - | *Sinorhizobium meliloti 1021* | 1.41 (696) | Q92RS8 | 26.11 | |
| **4L69 [12]** | - | *Mycobacterium tuberculosis* | 1.93 (60) | P9WGX1 | 27.35 | |
| **5O95 [18]** | - | *Legionella pneumophila* | 1.27 (880) | Q5ZRE6 | 36.97 | |
| **5O96 [18]** | SAM | *Legionella pneumophila* | 1.23 (828) | Q5ZRE6 | 36.97 | |
| **5VM8 [18] *** | SAM | *Neisseria gonorrhoeae* | Ref. (948) | Q5F4Y3 | 100 | |

* Unpublished, cf. Table S1.

*Molecular trajectory of SAH in the N. gonorrhoeae RsmE.*

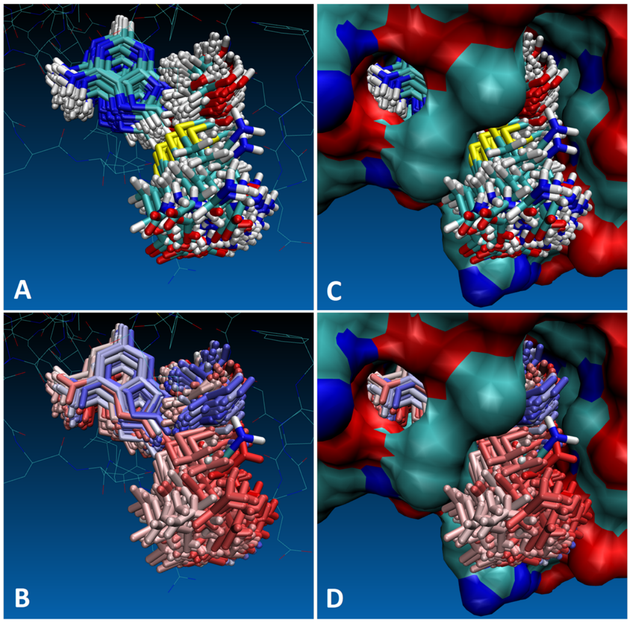


**Figure S3.** Molecular dynamics trajectories of SAH. (**A**) SAH trajectory colored by element. (**B**) SAH trajectory colored by simulation time-step. (**C**) SAH trajectory colored element inside surfaced

binding site, only backbone atoms colored by element. (**D**) SAH trajectory colored by simulation time-step inside the binding site surface. All trajectories were aligned with the VMD Trajectory tool and were tracked every 10 steps (over 10000). Color code for time-step trajectories (Bottom): Red trajectory steps are in the first third of simulation time (0 to 33 ns approx.), white trajectory steps belong to the second third of simulation time (33 to 66 ns approx.) and blue trajectory steps are in the last third of simulation time (66 to 100 ns approx.). All the trajectories are superimposed with the receptor in line (left) and solid surface (right) representations at time-step 0.

*SAM and SAH protonation states*

For SAM and SAH protonation states the crystal structures of SAM and SAH were inspected (5VM8 and 2EGW [12]). On the one hand, the amine group in the methionine/homocysteine tail may contact with Gly195. It is not directly exposed to water but surrounded by neutral to lipophilic side chains (Figure 15). Hence, its deprotonated state was assumed – regardless of the pKa values for the C-alpha ammonium group of SAM or SAH in pure water around 9.4 or 9.5 {60,61]. On the other hand, the C-alpha carboxyl group is exposed to the solvent without interacting with any other residue. Therefore, it was concluded that it was deprotonated due a pKa value ranging between of 1.7 and 1.8, respectively {60,61].

*Docking with the prevailing SAM cation.*

Molecular docking was conducted with the RsmE targets of N. gonorrhoeae and E. coli interacting with the amine group of SAM in the protonated state, showing binding energy values very close to those estimated for the non-protonated amino group (Table S3).

**Table S3.** Computed binding energies for monocationic SAM against target proteins of either *N. gonorrhoeae* or *E. coli* RsmE (**Table S4**). The energy differences (last column) are neglectable because they should be at least ten to hundred times higher to become significant under Autodock 4.2.

| **PDB file [Ref.] Species** | **Type** | **Ligand** | **SAM-NH$_2$ (non-protonated) $\Delta G_{binding}$ Kcal/mol** | **SAM-NH$_3^+$ (protonated) $\Delta G_{binding}$ Kcal/mol** | **$\Delta G_{binding}$ difference Kcal/mol** |
|---|---|---|---|---|---|
| **5VM8 [18] *** *N. gonohrroeae* | RsmE | SAM | - 8.9 | - 9.3 | 0.4 |
| **4E8B [2]** *E. coli* | RsmE | SAM | - 6.2 | - 6.0 | 0.2 |

* Unpublished, cf. Table S1.

*Energy minimization of the E. coli RsmE-ligand complex.*

As of April 2022, the crystal structure of *E. coli* RsmE in complex with SAM or SAH has not yet been solved, but the monomer has been solved by X-ray (PDB file 4E8B [2]). Both SAM and SAH ligands were docked in *E. coli* RsmE to compare them with the crystal complexes of *N. gonorrhoeae* and *A. aeolicus*.

The first molecular docking results of SAM and SAH with the crystal *E. coli* RsmE yielded very different binding energy values to those obtained with the other two species (approximately a thousand times lower). SAH and SAM complexed with *E. coli* RsmE by docking simulations are displaced by 2.3 and 2.8 Å respectively from their position observed in *A. aeolicus* and *N. gonorrhoeae*. This may be due to the fact that *E. coli* RsmE crystallized in the absence of a ligand or it is an artifact. Whatever the reasons might be, the *E. coli* RsmE binding site has a larger space compared to the other two binding sites analyzed in this work, and hence the docking results employing this crystal receptor are not comparable.

*Molecular docking with the E. coli RsmE crystal structure*

For *E. coli* RsmE, the initial docking results were considered not predictive because of the open state of the crystal RsmE binding site. In both docked ligands with *E. coli* RsmE, the methionine/homocysteine tail tended to roll over, mainly because of the breadth of the binding site is wider than that of other species. The interaction between the N (N2) amine of SAM and Pro195 was assumed to be random (Fig. S3).

SAH and SAM docked with the crystal RsmE are displaced by 2.3 and 2.8 Å from their position observed in *A. aeolicus* and *N. gonorrhoeae* structures respectively. This displacement caused the adenine ring to interact with Leu217, Gly218, and Arg220 at the loop between β12 and α6, albeit hindered all other possible interactions between the SAM methionine tail (homocysteine in SAH) and other conserved residues, that is Leu171 and Gly194. This hindrance might have led to a significant reduction in binding energies.

The interactions between the sugar hydroxyl (O2′) and Met169 in *N. gonorrhoeae* or Leu161 in *A. aeolicus* were regarded as fundamental because they were present in both species. Such interactions, however, could not be observed during docking simulations of *E. coli* RsmE and Leu171. The *E. coli* RsmE binding site clearly defined a zone of H-bond acceptor residues (Fig. S3, F, left side), which was very similar to other species that correspond to the backbone atoms of conserved Leu217, Gly218, and Arg220.

*Superposition of SAM and SAH within the RsmE binding site of the three species.*

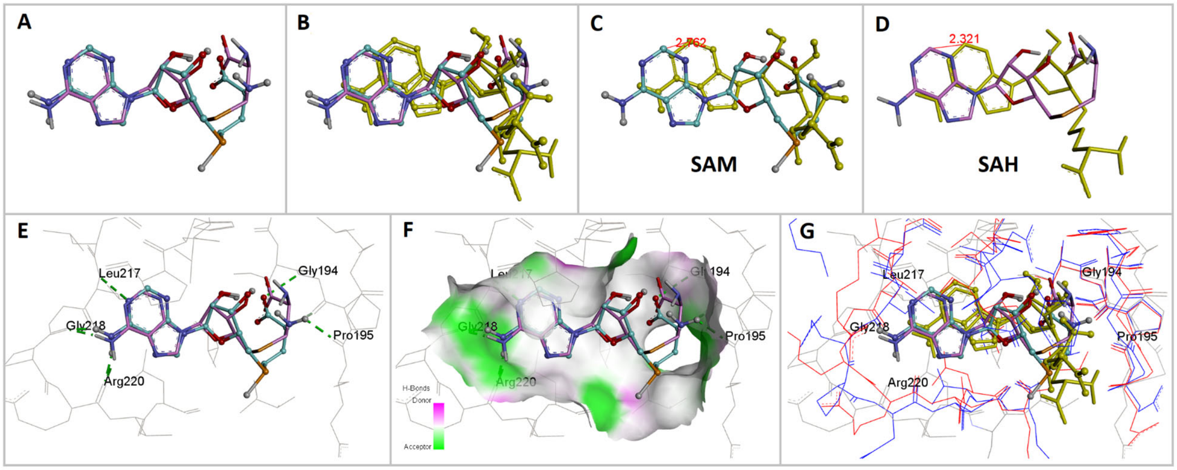


**Figure S4.** (**A**) Superposition of docked SAM and SAH (ball and stick, light blue carbons) and docked SAH conformation (stick, violet carbons) within the crystal binding site RsmE of *E. coli* (4E8B). (**B**) The docked conformations of SAM and SAH at the RsmE binding site of the crystal structure from *E. coli* in relation to the crystal structures of (**C**) SAM and (**D**) SAH from 5VM8 and 2EGW respectively (both in yellow). (**E**) Interactions of docked SAM and SAH conformations with residues of the crystal structure at the RsmE binding site of *E. coli* (4E8B). (**F**) Surface of the crystal *E. coli* binding site with potential zones for H-bonds formation interacting with docked SAM and SAH. (**G**) Docked SAM and SAH in *E. coli* RsmE *vs* crystal structures of SAM and SAH of *N. gonorrhoeae* and *A. aeolicus* from 5VM8 and 2EGW respectively (both in yellow) inside of the three superimposed RsmE binding sites of *N. gonorrhoeae* (red), *A. aeolicus* (blue), *E. coli* (gray) depicted as sticks. Distances are measured in Å.

By superimposing the active sites of the three species, it can be seen that the backbone of the loop between β11 and α5 and the loop between β12 and α65 in *E. coli* are more distant from the ligand than in the *N. gonorrhoeae* RsmE. In the bottom-right image of Fig. S4, the gray lines that correspond to the *E. coli* backbone binding site are more separated from the ligands and from each other than the red and blue lines from *N. gonorrhoeae* and *A. aeolicus* respectively.

*Interaction matrix of the binding sites of the RsmE of the three species.*

**Table S4.** Interaction matrix for SAM and SAH with RsmE of *N. gonorrhoeae* (Ng), *A. aeolicus* (Aa), *E. coli* (Ec). rx: crystal structure, d: docked conformation. The binding energy of the crystal structure is not determined directly by Autodock (---). BdD: Blind dock, BkD: Backdock.

| **Ligand/PDB_Specie**<br>$\Delta G_{binding}$ (Kcal/mol) | **Interacting residue** | | | | | | | | |
|---|---|---|---|---|---|---|---|---|---|
| **SAM_rx/5VM8_Ng**<br>--- | **Met169** | **Gly192** | **Pro193** | **Glu194** | **Gly195** | **Leu215** | **Gly216** | **Arg218** | **Leu220** |
| **SAM_d/5VM8_Ng**<br>- 8.91 (BkD) | | | | | | | | | |
| **SAH_d/5VM8_Ng**<br>- 8.67 (BdD) | | | | | | | | | |
| **SAH_rx/2EGW_Aa**<br>--- | **Leu161** | **Gly185** | **Pro186** | **Glu187** | **Gly188** | **Leu208** | **Glu209** | **Tyr211** | **Leu213** |
| **SAH_d/2EGW_Aa**<br>- 9.65 (BkD) | | | | | | | | | |
| **SAM_d/2EGW_Aa**<br>- 9.37 (BdD) | | | | | | | | | |
| **SAM_d/4E8B_Ec**<br>- 6.18 (BdD) | **Leu171** | **Gly194** | **Pro195** | **Glu196** | **Gly197** | **Leu217** | **Gly218** | **Arg220** | **Leu222** |
| **SAH_d/4E8B_Ec**<br>- 5.38 (BdD) | **Leu171** | **Gly194** | **Pro195** | **Glu196** | **Gly197** | **Leu217** | **Gly218** | **Arg220** | **Leu222** |

*Evaluation of the docking results*

The inhibitory constants of SAM and SAH against RsmE were taken from the PDB entries (5O95, 5O96 [18]). NpmA was also used as reference (PDB entries: 3P2E, 3P2K, and 3PB3) with inhibitory values from MOAD and PDBbind-CN databases {62-64]). Docking reliability was assessed under the settings described in the Method section. Docking was conducted with both ligands against their corresponding liganded crystal structures RsmE (*back docking* of two ligands: SAH against 2EGW for *A. aeolicus* and SAM against 5VM8 for *N. gonorrhoeae*). The calculated binding energy values ($\Delta G_{binding}$) of the docked conformation closest to the crystal ligand positions are shown in Table S5 along with previously reported values. Gibbs free binding energy was calculated from the reported $K_i$ or $K_d$ constant (or vice versa for AutoDock Tools) using the $\Delta G° = -RT\ln K_i$ *(or* $K_d$*)* equation at 298 K [65].

**Table S5.** Reported values for inhibitory constants (Ki) from Binding-MOAD (the Mother of All Databases) and PDBbind-CN databases of different methyltranferases. AD: AutoDock version 4.2. $^{m}$: mutations in Leu31Met, Leu90Met, Leu128Met, Leu196Met; Kd : dissociation constants ; Ka: affinity contants

| **PDB file [Ref.] Species** | **Type** | **Ligand** | **MOAD* or PDBbind⁺ Ki, Ka, Kd values** | **MOAD or PDBbind $\Delta G_{binding}$ Kcal/mol** | **Docking (AD) $\Delta G_{binding}$ Kcal/mol** | **Docking (AD) constant value** | **$\Delta G_{binding}$ absolute difference Kcal/mol** |
|---|---|---|---|---|---|---|---|
| **5O96 [18]**<br>*L. pneumophila* | RsmE | SAM | *Ka = 708000 $M^{-1}$<br>Ki = 1.41 µM | - 8.0 | - 7.8 | Ki = 1.83 µM | 0.2 |
| **3P2K [62]**<br>*E. coli* | NpmA | SAM | ⁺Kd = 20000 nM | - 6.4 | - 9.8 | Kd = 65 nM | 3.4 |
| **3P2E [62]**<br>*E. coli* | NpmA | SAH | *⁺Kd = 600 nM | - 8.5 | - 9.3 | Kd = 162 nM | 0.8 |

| | | | | | | | |
|---|---|---|---|---|---|---|---|
| **3PB3[m] [62]** *E. coli* | NpmA | SAH | [+]Kd = 600 nM | - 8.5 | - 8.9 | Kd = 294 nM | 0.4 |
